# 1例*EGFR*驱动基因阳性晚期肺癌患者的综合治疗

**DOI:** 10.3779/j.issn.1009-3419.2021.101.20

**Published:** 2021-06-20

**Authors:** 帅 王, 先军 闵, 影顺 杨, 国田 裴, 强 刘, 军 刘, 宇清 黄

**Affiliations:** 100080 北京，北京市海淀医院（北京大学第三医院海淀院区）胸外科 Department of Thoracic Surgery, Beijing Haidian Hospital (Haidian Section of Peking University Third Hospital), Beijing 100080, China

**Keywords:** 肺肿瘤, 表皮生长因子受体酪氨酸激酶抑制剂, 不良反应, Lung neoplasms, Eepidermal growth factor receptor-tyrosine kinase inhibitor, Adverse reaction

## Abstract

肺癌的发病率和病死率居全球恶性肿瘤首位，部分患者就诊时已发生远处转移，严重影响生存期及生活质量。随着分子靶向药物的发展，肺癌治疗方式发生变革，精准医疗模式应用临床，不但延长了患者生存期，而且提高了生活质量。本文回顾性分析北京市海淀医院胸外科5年前收治的1例晚期肺癌患者，依据基因检测及循环肿瘤DNA（circulating tumor DNA, ctDNA）变化选择合理治疗方案，并对治疗过程的不良反应进行总结。患者女性，5年前确诊为Ⅳ期表皮生长因子受体（epidermal growth factor receptor, *EGFR*）驱动基因阳性的非小细胞肺癌，通过靶向药物、靶向药物联合化疗、靶向药物联合抗血管生成药物以及转移灶的放疗，延长生存时间，提高生活质量。本文中*EGFR*驱动基因阳性的晚期非小细胞肺癌患者，基于精准基因分型以及动态监测，进行个体化治疗，在控制肿瘤的同时，毒副作用反应较为温和。患者生存期得到延长，生活质量得到保证。

## Abstract (en)

Lung cancer is the most common malignant tumor and the leading cause of cancer-related death worldwide. Most of the patients have distant metastasis when visiting the doctor, which seriously affects the survival time and quality of life of the patients. With the development of molecular targeted drugs, lung cancer treatment has been transformed from traditional chemotherapy to targeted therapy and precision medicine has been gradually applied in clinical practice, which can make lung cancer patients live longer and have a better quality of life. We present a case of advanced lung cancer patient who presented to Department of Thoracic Surgery of Beijing Haidian Hospital five years ago. We chose the reasonable treatment options though the genetic tests and circulating tumor DNA tests. We summarized the adverse reactions in the whole course of treatment. The comprehensive therapy we utilized, including targeted therapy, chemotherapy, antiangiogenic agents and local radiotherapy, have resulted in our patient with remaining alive. For advanced non-small cell lung cancer with epidermal growth factor receptor (*EGFR*) mutation positive, individualized treatment was conducted based on precise genotyping and dynamic monitoring, which can not only control the tumor, but also have mild toxic and side effects. The survival time of the patients was prolonged and the quality of life was guaranteed.

## 1 病例资料

患者，女性，47岁，因“左侧胸痛”于2015年10月14日入院。既往无吸烟病史，肿瘤家族史：父亲淋巴瘤。外院胸部计算机断层扫描（computed tomography, CT）（2015年10月8日）可见：右肺下叶软组织阴影，大小约2.6 cm×1.5 cm（图 1A），左侧第6肋骨骨质破坏。胸椎磁共振成像（magnetic resonance imaging, MRI）（2015年10月23日，北京市海淀医院）提示：胸骨及胸4/6/7/10/11椎体及附件多发软组织信号影，符合胸骨、胸腰椎多发转移。肿瘤标记物明显升高：癌胚抗原（carbohydrate antigen, CEA）：89.8 ng/mL，糖类抗原199（carbohydrate antigen 199, CA199）：175.8 U/mL，细胞角蛋白19片段（cytokeratin-19-fragment, Cyfra21-1）：3.80 ng/mL。2015年10月15日行支气管内超声引导针吸活检术（endobronchial ultrasonography transbronchial needle asprtaion, EBUS-TBNA），病理提示：送检组织中可见少量异型细胞，免疫组化染色结果提示：甲状腺转录因子-1（thyroid transcription factor-1, TTF-1）（+）、CK7（+）、CEA（+）、NapsinA（+）、CK5/6（-）、p40（-）、p63（-）、Syn（-）、CD56（-）、Ki-67（30%），考虑腺癌可能性大。基因检测（139个基因）：表皮生长因子受体（epidermal growth factor receptor, EGFR）基因：外显子19突变，突变频率：78.18%。*PIK3CA*基因：外显子20突变，突变频率：41.8%。初步诊断为：右肺腺癌，T1bN2M1b Ⅳ期，*EGFR*突变阳性，体能状态（performance status, PS）评分为2分。

**图 1:**
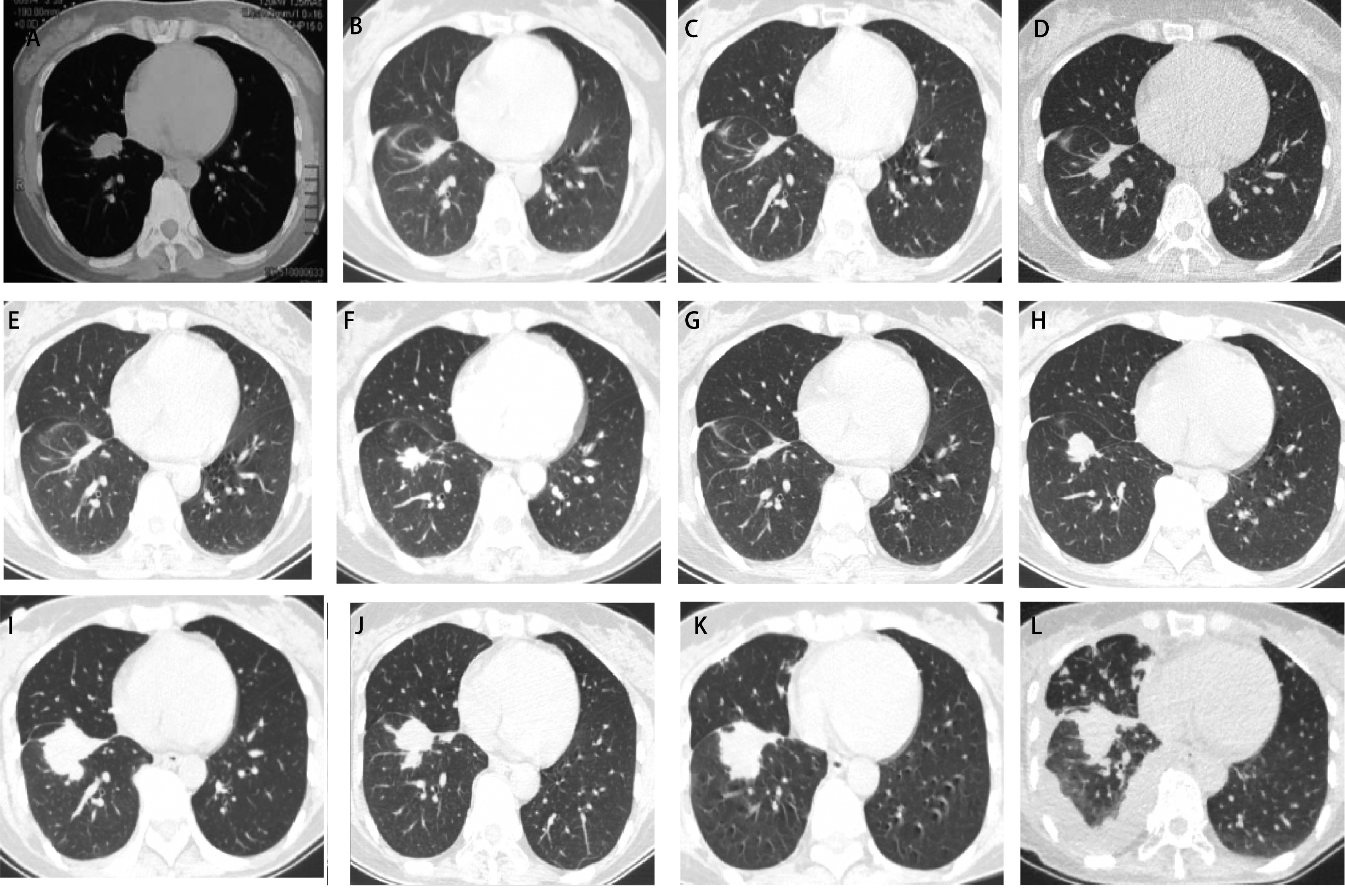
胸部CT。治疗前（A）、吉非替尼治疗3个月（B）、吉非替尼治疗12个月（C）、奥希替尼治疗10个月（D）、化疗2周期后（E）、头颅放疗后（F）、单药培美曲塞2个周期后（G）、股骨放疗后（H）、安罗替尼联合奥希替尼治疗4个月（I）、恢复靶向药物治疗后3个月（J）、恢复靶向药物治疗5个月（K）、营养支持期间（L）的胸部CT情况。 Chest CT. Pre-treatment (A), Gefitinib for 3 months (B), Gefitinib for 12 months (C), Osimertinib for 10 months (D), after 2 cycles of chemotherapy (E), after cranial radiotherapy (F), after 2 cycles only pemetrexed (G), after femur radiotherapy (H), Anlotinib and Osimertinib for 4 months (I), after 3 months of resumption targeted drug (J), after 5 months of resumption targeted drug (K), period of nutritional support (L) of chest CT. CT: computed tomography.

初始治疗方案为：一代表皮生长因子受体酪氨酸激酶抑制剂（epidermal growth factor receptor-tyrosine kinase inhibitors, EGFR-TKIs）：吉非替尼联合局部肋骨放疗、双磷酸盐治疗。口服吉非替尼3个月，疗效评价为部分缓解（partial response, PR）（图 1B）。口服吉非替尼第8个月时对比治疗前后，骨质破坏修复情况（图 2）。至口服吉非替尼第12个月（图 1C）肺部疾病稳定（stable disease, SD），肿瘤标记物：CEA：63.52 ng/mL，CA199：138.3 U/mL，Cyfra21-1：2.02 ng/mL。此时患者腰部疼痛剧烈，影像学提示腰椎骨质破坏。复测循环肿瘤DNA（circulating tumor DNA, ctDNA）结果提示：*EGFR* 19del突变（丰度5%），T790M突变（丰度1%），*PIK3CA*基因：外显子20（丰度2%）。

**图 2:**
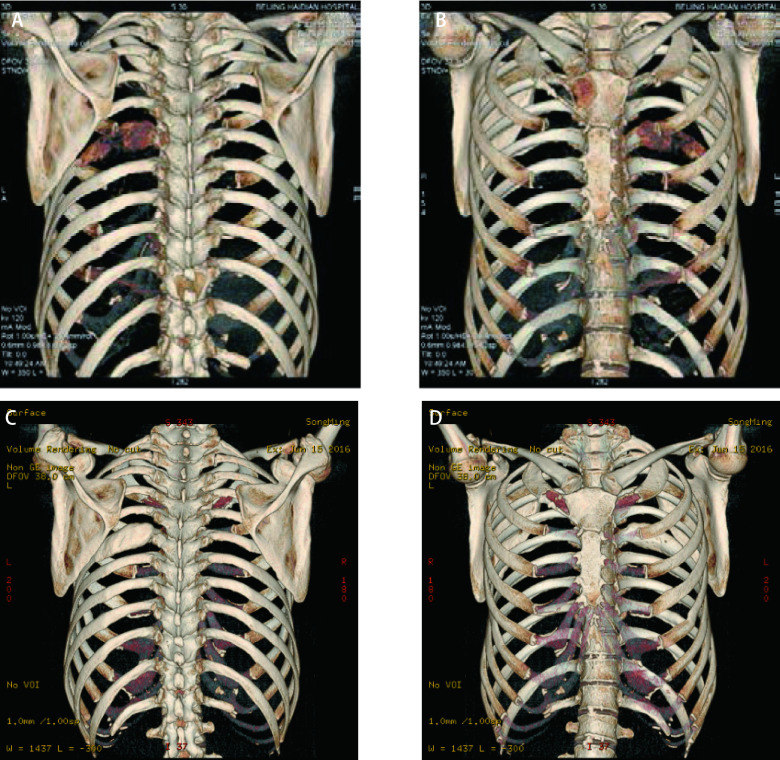
胸廓的三维成像。治疗前：左侧肋骨（A）及胸骨柄右侧（B）骨破坏明显；治疗后：左侧肋骨（C）及胸骨柄右侧（D）骨破坏修复。 3D chest imaging. Before treatment: bone destruction of left rib (A) and lateral border manubrium (B); after treatment: bone destruction of left rib (C) and lateral border manubrium (D) was repaired.

二线治疗方案为：奥希替尼联合腰椎放疗、双膦酸盐治疗。PS评分为2分。患者外院行腰椎放疗后腰痛症状改善。口服奥希替尼4月余肺部病变为SD，口服奥希替尼8月余右肺下叶病变缓慢增大，至2017年8月，肺内病变明显增大（图 1D），出现疾病进展（progressive disease, PD）。复测ctDNA未见阳性突变。

三线治疗方案为：吉非替尼联合培美曲塞（860 mg）/顺铂（120 mg）。PS：2分，身高：170 cm，体重：62 kg，肿瘤标记物：CEA：3.34 ng/mL，CA199：32.81 U/mL，Cyfra21-1：1.17 ng/mL。含铂双药化疗2个周期后肺部病变呈PR（图 1E），直至6个周期结束后肺内病变稳定，后续单药维持2个周期肺部病变稳定，后续出现化疗相关性肺炎，以及肝功能异常，因此暂停化疗，抗炎、保肝治疗，吉非替尼维持治疗，定期复查胸部CT肺部病变稳定。2018年7月17日复查头颅MRI平扫未见转移瘤。直至2018年10月头颅CT发现颅内多发转移瘤，出现PD。

后线治疗方案为：奥希替尼+头颅放疗+贝伐珠单抗治疗。PS：3分。全脑放疗后颅内肿瘤控制，水肿消失，中线居中，患者临床症状改善。就在此时肺内病变出现增大（图 1F），疗效为PD。

后线治疗方案：奥希替尼+培美曲塞单药（860 mg）+贝伐珠单抗（470 mg）。PS：1分，身高：170 cm，体重：62.5 kg。距离上一次培美曲塞间隔1年，且上次使用培美曲塞过程中对肺部病变控制好，因此继续使用单药培美曲塞。治疗2个周期评估疗效PR（图 1G），4个周期疗效为SD。在6个周期结束后患者出现右侧髋关节疼痛明显、跛行，骨扫描提示右侧股骨病变较前明显进展。针对右侧股骨转移灶外院放疗科会诊行局部放疗，放疗期间口服奥希替尼治疗。放疗后复查胸部CT提示肺内病变再一次出现进展（图 1H），此时头颅MRI颅内转移瘤同前，ctDNA仍为阴性。外院多学科会诊后看可考虑加用安罗替尼。

后线治疗方案为：奥希替尼联合安罗替尼。口服安罗替尼2个月病变稳定。再过1个月余肺部病变增大，肿瘤标记物：CEA：3.66 ng/mL，CA19-9：25.30 U/mL，Cyfra21-1：3.69 ng/mL，神经元特异烯醇化酶（neuronspecific enolase, NSE）：12.84 ng/mL。疗效评估：PD。多线治疗后肿瘤进展，担心存在病理类型转化。于2020年1月2日行CT引导下穿刺活检及射频消融术。穿刺病理结果为：可见少量巢状分布的肿瘤浸润，细胞显著异型，免疫组化染色结果：TTF-1（+）、CK7（+）、ALK-Ventana（-）、CK5/6（-）、p40（-）、Ki-67（50%），符合腺癌。基因检测结果提示：*EGFR*外显子19非移码缺失突变，丰度为11.6%。继续让患者口服奥希替尼联合安罗替尼治疗，1个月后胸部CT显示肺部病变增大，出现PD（图 1I）。2020年2月初-3月17日因疫情爆发，患者擅自停用靶向药物，考虑肿瘤进展可能与停用靶向药物有关。根据前期基因检测结果，让患者恢复奥希替尼联合安罗替尼治疗。1个月后复查胸部CT病情稳定，3个月再次复查胸部CT（图 1J）疗效PR。2020年8月20日因“间断咳嗽及消瘦”入院检查，胸部CT（图 1K）提示右肺下叶病变及转移灶增大。肿瘤标记物明显升高：CEA：28.93 ng/mL，CA199：28.66 U/mL，Cyfra21-1：4.18 ng/mL，NSE：18.83 ng/mL。考虑肿瘤进展，但患者入院前1个月间断腹泻，间断口服益生菌及止泻药物，体重下降明显，因此针对腹泻对症治疗，并进行营养支持。此时头颅MRI提示颅内病变同前。2020年10月8日患者咳嗽加重，伴胸闷憋气不适，复查胸部CT（图 1L）可见右侧胸腔积液，出现PD。再次就诊我科，计划行下一步抗肿瘤治疗，目前行白蛋白紫杉醇单药联合奥希替尼治疗。此时因其反复鼻出血、痰中带血以及高血压副作用，停用安罗替尼，目前患者诊治仍在继续。

回顾患者整个治疗过程中不良反应：采用EGFR-TKIs治疗期间对于不良反应预防为主，对症治疗：皮肤方面：防晒，保持皮肤的清洁与湿润，可适当给润肤露涂抹；饮食方面：低脂低纤维饮食，清洁饮食，不得随意服用泻药；定期监测肝功能等。

抗血管生成药物不良反应：高血压、蛋白尿、出血、消化道穿孔以及血栓等。2020年8月住院期间出现高血压，最高达160 mmHg/109 mmHg，心内科会诊给予钙离子通道阻滞剂后血压控制稳定，后反复出现鼻出血以及痰中带血，因此停用安罗替尼。

## 2 讨论

原发性肺癌是当前威胁着人类生命健康的恶性肿瘤之一，其发病率及死亡率在我科乃至国外逐年上升，目前其发病率和死亡率居恶性肿瘤首位^[1, 2]^，其中非小细胞肺癌（non-small cell lung cancer, NSCLC）占85%以上。早期肺癌发病隐匿，50%左右在初诊时为晚期，骨转移是主要的血运转移部位^[3]^。肺癌骨转移发生率为10%-15%^[4]^，骨转移常常预示患者的生存期及生活质量均受到严重威胁，经过治疗后1年的生存率为40%-50%，肺癌骨转移的好发部位在脊柱和躯干骨近端，发生于脊柱者约占50%，股骨占25%，肋骨和胸骨占12%^[5, 6]^。肺癌骨转移的治疗目标是提高生活质量、延长生命、缓解症状及心理痛苦、预防或延缓病理性骨折等骨相关事件（skeletal related events, SREs）。

EGFR-TKIs治疗：随诊分子分型的进展，晚期肺癌的治疗模式发生转变，2009年发表的研究——IPASS，Ⅲ期随机试验，证明了EGFR-TKIs治疗敏感突变患者的有效性，*EGFR*外显子19或21突变的肿瘤患者能从吉非替尼中获益显著，而野生型的EGFR患者与化疗相比无进展生存期（progression-free survival, PFS）明显较差。*EGFR*突变患者中，初治方案选择吉非替尼优于卡铂-紫杉醇^[7]^。*EGFR*突变阳性晚期NSCLC患者一线治疗采用TKI：厄洛替尼、埃克替尼及阿法替尼对比化疗均可显著改变患者的PFS，且不良反应显著低于化疗^[8, 9]^。EGFR-TKIs在世界范围内已被证实在晚期NSCLC伴有*EGFR*敏感突变类型的患者中疗效显著^[10, 11]^，因此指南也明确推荐EGFR-TKIs作为*EGFR*晚期突变腺癌的首选方案。

EGFR-TKIs耐药：但其在延长PFS同时其耐药突变也需要考虑，一般于1年左右出现，其中50%患者会出现*EGFR* T790M突变。*EGFR* T790M基因突变与初始治疗前是否发生骨转移以及与EGFR-TKIs敏感性有关^[12, 13]^。此时ctDNA打破了创伤性的活检，并且在临床实践中扮演重要角色，其特异性较高（97.2%-100%），但其灵敏度报道不一。血液是*EGFR*基因突变检测合适的替代标本，能够有效指导临床用药。三代TKIs：奥希替尼作用于T790M该靶点，为耐药患者带来希望。AURA3 Ⅲ期临床研究，在一线EGFR治疗失败后，出现T790M突变的晚期NSCLC中，对比奥希替尼和含铂化疗联合培美曲塞的疗效及安全性，结果显示奥希替尼效果显著。伴有脑转移的患者中枢神经系统客观缓解率为70%，而化疗组仅为31%；PFS显著延长（11.7个月*vs* 5.6个月）^[14]^。奥希替尼已被指南列为一线治疗以及治疗失败后出现T790M突变的治疗方式。

联合治疗：EGFR联合化疗：Cheng等^[15]^一项Ⅱ期试验中得出，吉非替尼联合培美曲塞组中位PFS为15.8个月，而单药吉非替尼为10.9个月，组间差异显著（*P*=0.029）。EGFR-TKIs联合抗血管生成药物：Ⅱ期的J025567研究^[16]^显示贝伐珠单抗联合厄洛替尼与单药厄洛替尼相比一线治疗治疗晚期*EGFR*阳性患者，除外脑转移患者，中位PFS在联合治疗组与单药组间差异显著（16.0个月*vs* 9.7个月，*P*=0.001, 5）。一线贝伐珠单抗联合厄洛替尼以及安慰剂联合厄洛替尼相比试验CTONG1509^[17]^显示，贝伐珠单抗联合厄洛替尼以及安慰剂联合厄洛替尼组的中位PFS有统计学差异（18.0个月*vs* 11.3个月，*P* < 0.001），并且验证了贝伐珠单抗联合厄洛替尼在中国人群的安全性。

药物不良反应：EGFR-TKIs药物不良反应可累及全身各个系统，最常见的药物不良反应为腹泻和皮肤反应，大多数是可逆，一般见于服药后的第1个月内；间质性肺炎、急性肺损伤以及急性心梗等罕见不良反应的预后差，临床因引起重视。临床上因在使用药物前宣教、预防，做到早发现、早处理，预防严重及死亡事件发生。一代TKIs：吉非替尼、厄洛替尼、埃克替尼在多项研究中其腹泻总发生率为9.5%-57%，≥3级的发生率为0.9%-7.4%；皮疹/痤疮痒皮疹发生率为14.9%-73%，≥3级的发生率为2%-14.9%；转氨酶升高发生率为6%-55.3%，≥3级的发生率为2%-26.3%。二代TKIs：阿法替尼腹泻发生率为88.3%-95.2%，≥3级的发生率为5.4%-14.4%；皮疹/痤疮痒皮疹发生率为80.8%-89.1%，≥3级的发生率为14.6%-16.2%；转氨酶升高发生率为15.1%-20.1%，≥3级的发生率为0.4%-1.7%。三代TKIs：奥希替尼腹泻发生率为41.0%，≥3级的发生率为1.0%；皮疹/痤疮痒皮疹发生率为34%，≥3级的发生率为1%；转氨酶升高发生率为1%-2%，≥3级的发生率为1%^[7-9, 18-22]^。其中间质性肺疾病发生率较低，但其发展迅速，病死率高，因此早期识别并采取措施尤为重要。

总之，EGFR-TKIs改善晚期NSCLC患者生存期的同时，需要监测其不良反应，其中大部分的不良反应是可控的。因此对于使用EGFR-TKIs前，应对其进行服药前宣教，服药期间做好不良反应监测，早期识别，早期处理，避免影响药物中断。这样既能延长患者生命长度，也能拓展生命宽度。
